# Pancreatic cancer-associated gene polymorphisms in a nation-wide cohort of p16-*Leiden* germline mutation carriers; a case–control study

**DOI:** 10.1186/s13104-015-1235-4

**Published:** 2015-06-26

**Authors:** Thomas P Potjer, Nienke van der Stoep, Jeanine J Houwing-Duistermaat, Ingrid C A W Konings, Cora M Aalfs, Peter C van den Akker, Margreet G Ausems, Charlotte J Dommering, Lizet E van der Kolk, Merel C Maiburg, Liesbeth Spruijt, Anja Wagner, Hans F A Vasen, Frederik J Hes

**Affiliations:** Department of Clinical Genetics, Leiden University Medical Center, Albinusdreef 2, 2333 ZA Leiden, The Netherlands; Department of Medical Statistics and Bioinformatics, Leiden University Medical Center, Leiden, The Netherlands; Department of Gastroenterology and Hepatology,Erasmus MC, University Medical Center, Rotterdam, The Netherlands; Department of Clinical Genetics, Academic Medical Center, Amsterdam, The Netherlands; Department of Genetics, University of Groningen, University Medical Center Groningen, Groningen, The Netherlands; Department of Medical Genetics, University Medical Center Utrecht, Utrecht, The Netherlands; Department of Clinical Genetics and Human Genetics, VU University Medical Center, Amsterdam, The Netherlands; Department of Clinical Genetics, The Netherlands Cancer Institute, Amsterdam, The Netherlands; Department of Clinical Genetics, Maastricht University Medical Center, Maastricht, The Netherlands; Department of Clinical Genetics, Radboud University Medical Centre, Nijmegen, The Netherlands; Department of Clinical Genetics, Erasmus MC, University Medical Center, Rotterdam, The Netherlands; The Netherlands Foundation for the Detection of Hereditary Tumours, Leiden, The Netherlands

**Keywords:** CDKN2A, p16-Leiden, Germline mutation, FAMMM syndrome, Pancreatic cancer, Modifiers, Single nucleotide polymorphisms

## Abstract

**Background:**

The p16-*Leiden* founder mutation in the *CDKN2A* gene is the most common cause of Familial Atypical Multiple Mole Melanoma (FAMMM) syndrome in the Netherlands. Individuals with this mutation are at increased risk for developing melanoma of the skin, as well as pancreatic cancer. However, there is a notable interfamilial variability in the occurrence of pancreatic cancer among p16-*Leiden* families. We aimed to test whether previously identified genetic risk factors for pancreatic cancer modify the risk for pancreatic cancer in p16-*Leiden* germline mutation carriers.

**Methods:**

Seven pancreatic cancer-associated SNPs were selected from the literature and were genotyped in a cohort of 185 p16-*Leiden* germline mutation carriers from 88 families, including 50 cases (median age 55 years) with pancreatic cancer and 135 controls (median age 64 years) without pancreatic cancer. Allelic odds ratios per SNP were calculated.

**Results:**

No significant association with pancreatic cancer was found for any of the seven SNPs.

**Conclusions:**

Since genetic modifiers for developing melanoma have already been identified in *CDKN2A* mutation carriers, this study does not exclude that genetic modifiers do not play a role in the individual pancreatic cancer risk in this cohort of p16-*Leiden* germline mutation carriers. The search for these modifiers should therefore continue, because they can potentially facilitate more targeted pancreatic surveillance programs.

## Background

The melanoma gene *CDKN2A* produces two important proteins: p16^INK4a^, which is a cyclin-dependent kinase inhibitor, and p14^ARF^, which binds the p53-stabilizing protein MDM2 [1]. In the Netherlands, a founder mutation in the *CDKN2A* gene, a 19-base pair deletion called p16-*Leiden* (c.225_243del19; RefSeq NM_000077.4), is the most common cause of Familial Atypical Multiple Mole Melanoma (FAMMM) syndrome [2]. In addition to a marked increased risk of developing melanoma of the skin (70% lifetime risk), these mutation carriers also have a 15–20% lifetime risk of developing pancreatic cancer with a mean age of 58 years at diagnosis [3]. Interestingly, there is a notable interfamilial variability in the occurrence of pancreatic cancer among p16-*Leiden* families [3]. Therefore, the p16-*Leiden* mutation might not be the only genetic risk factor in these individuals causing an increased susceptibility for pancreatic cancer. Since pancreatic cancer has a very poor prognosis due to late occurrence of symptoms and therefore late detection, surveillance for pancreatic cancer is currently offered to p16-*Leiden* germline mutation carriers in a research setting to investigate whether pancreatic cancer, or, even more preferable, high-grade precursor lesions can be detected earlier in a potentially still curable stage [4]. By identifying additional genetic risk factors (genetic modifiers) in these individuals, surveillance could possibly be more individualized.

In recent years, genome-wide association studies (GWAS) have identified several common risk variants associated with pancreatic cancer [5–7]. In this study, we genotyped a selected number of these variants (SNPs) in a unique cohort of p16-*Leiden* mutation carriers with and without pancreatic cancer. We hypothesized that these SNPs might modify the risk of pancreatic cancer in these p16-*Leiden* mutation carriers.

## Methods

### Study population and DNA sample collection

For this case–control study, only proven carriers of the p16-*Leiden* germline mutation were included. From all Dutch p16-*Leiden* mutation carriers, DNA is stored in the Laboratory for Diagnostic Genome Analysis (LDGA) of the Leiden University Medical Center. All p16-*Leiden* germline mutation carriers diagnosed at the LDGA between the initiation of *CDKN2A* gene diagnostics at the LDGA in 1998 and January 1st 2014 were eligible for inclusion. Cases were defined as having been diagnosed with exocrine pancreatic cancer at the time of data collection; controls were at least 55 years old on January 1st 2014 or died beyond that age, and were not diagnosed with pancreatic cancer. Individuals who were younger than 55 years or died before this age were excluded from the control group. Medical records were obtained for each individual from the electronic hospital information system of the medical center where this individual initially received genetic counselling by a clinical geneticist for *CDKN2A* gene diagnostics. Access to these medical records was granted for the (co)authors since they are clinical geneticists working in these medical centers. Additional follow up data was acquired from two ongoing pancreatic surveillance studies (a single-center study at Leiden University Medical Center and a multi-center study at Erasmus MC University Medical Center Rotterdam) and from the Netherlands Foundation for the Detection of Hereditary Tumours, a central registration institute for hereditary tumours (FAMMM, amongst others) in the Netherlands. This study was approved by the Ethics Committee of the Leiden University Medical Center, by issuing a *declaration of no objection* (#P14.148). This is an assessment of the study protocol on due diligence, that is, if it serves the codes of *good practice* and *good conduct*. It is not a formal ethical assessment, because the study does not fall within the scope of the Dutch law for medical research on human subjects; the medical records and the DNA were already available and the involved human subjects were not specifically recruited for the study and were not subjected to any actions. Therefore, no separate ethical assessment or approval was needed for the collection of data in the participating medical centers.

### SNP selection and genotyping

SNPs for genotyping in this cohort were selected from recent GWAS studies with large cohorts of sporadic pancreatic cancer patients [5–7]. Selection was based on significance of association with pancreatic cancer and reported odds ratios, as well as expected allele frequencies. In the first place, SNPs with the largest odds ratios and smallest p-values were selected. Subsequently, only those SNPs with a relatively high minor allele frequency (MAF) were considered for genotyping, because of the limited sample size of the cohort. This would optimize the number of carriers of the minor allele and thereby augment the potential of reaching significance between subgroups. In order to test a relatively wide variety of genes, a maximum of two different SNPs per gene was maintained. All included individuals were genotyped for the selected SNPs using high-resolution DNA melting curve analysis [8]. Melting assays were performed with Lightscanner^®^ (Biofire Defense Inc, Salt Lake City, UT, USA).

### Statistical analysis

Given our relatively small sample size, we performed a power calculation with Bonferroni correction for multiple testing prior to the study. Despite a calculated power of approximately 15%, we wanted to pursuit this small but tangible chance of finding a trend of association.

The frequencies of the risk alleles were computed and compared between cases and controls by calculating a SNP-specific allelic odds ratio, including the 95% confidence interval. Additional p-values were calculated using a basic χ^2^ test and the Bonferroni correction for multiple testing was applied. Therefore, a *p* value of less than 0.007 was considered significant. All statistical analyses were performed using SPSS 20.0.0 (IBM Inc, Armonk, NY, USA).

## Results

### Patient characteristics (Table 1)

In total, 422 p16-*Leiden* germline mutation carriers were eligible for inclusion. Of these, 50 individuals (18 males, 36%) were diagnosed with pancreatic cancer, with a median age at diagnosis of 55 years (range 21–76 years), and could all be included in the case group. The remaining 372 individuals were not diagnosed with pancreatic cancer, but 237 individuals were excluded from the control group because of not having reached the age of 55 years. The remaining control group consisted of 135 individuals (50 males, 37%) with a median age of 64 years (range 55–88 years). Thus, for this study a total of 185 p16-*Leiden* germline mutation carriers (from 88 families) were included. A considerable number of individuals (98 of 185 [53%]) developed melanoma, and 37 of 98 individuals with melanoma (38%) had multiple melanoma. In the case group, 24 of 50 individuals (48%) had a medical history of melanoma, and in the control group 74 of 135 individuals (55%) had a medical history of melanoma.

**Table 1:** Patient characteristics

	p16-*Leiden* mutation carriers (N = 185)	
	Cases with pancreatic cancer (N = 50)	Controls without pancreatic cancer (N = 135)
Median age in years (range)	55 (21–76)	64 (55–88)
Gender (m:f)	18:32	50:88
Medical history of melanoma	24 (48%)	74 (55%)
*Of which multiple*	8/24 (33%)	29/74 (39%)

### SNP genotyping and association with pancreatic cancer

A total of seven SNPs in five different genes were selected for genotyping. Table 2 shows the minor allele frequencies of these SNPs in cases and controls, and the calculated association (allelic odds ratio and 95% confidence interval) with pancreatic cancer in our cohort of p16-*Leiden* germline mutation carriers. No significant association with pancreatic cancer was found for any of the seven SNPs.

**Table 2:** Association of seven selected SNPs with pancreatic cancer in this study

Marker^a^	Minor Allele Frequency		Allelic OR (95% CI)	Sign. (p value)
	Cases	Controls		
rs2821367 (T, C) 1q32.1 NR5A2	0.37	0.38	1.0 (0.62–1.62)	1.00
rs7310409 (G, A) 12q24.31 HNF1A	0.35	0.39	0.87 (0.54–1.41)	0.57
rs735396 (A, G) 12q23.31 HNF1A	0.33	0.36	0.90 (0.55–1.47)	0.67
rs1805100 (G, A) 8q21.11 HNF4G	0.49	0.56	0.76 (0.48–1.21)	0.24
rs505922 (T, C) 9q34.2 ABO	0.30	0.33	0.87 (0.53–1.44)	0.59
rs657152 (G, T) 9q34.2 ABO	0.30	0.37	0.73 (0.44–1.20)	0.22
rs172310 (C, A) 7q36.3 SHH	0.43	0.39	1.18 (0.74–1.89)	0.49

^a^SNP reference (major/minor allele), chromosome location, gene.

## Discussion

In this case–control study, we analysed seven pancreatic cancer-associated SNPs in a distinctively homogeneous cohort of 185 *CDKN2A* germline mutation carriers. That is, all cases with (n = 50) and all controls without (n = 135) pancreatic cancer carry the same p16-*Leiden* germline mutation. We hypothesized that (a subset of) these SNPs would be associated with an increased pancreatic cancer risk in these individuals, and that genetic modifiers would explain, at least partially, the variability in the occurrence of pancreatic cancer in p16-*Leiden* families. However, in our cohort, no significant association was found between the occurrence of pancreatic cancer and any of the seven SNPs.

Currently, research on genetic modifiers in *CDKN2A* mutation carriers is mainly focused on identifying low-risk variants that influence melanoma risk [9, 10]. To date, the MC1R gene, which is known to have a role in the skin pigmentation process, is the most important modifier gene identified so far [11, 12]. Penetrance of *CDKN2A* mutations regarding melanoma risk is thus proven to be subject to low-risk genetic variants in other genes. Because of these encouraging results from previous studies, it can be expected that genetic variants could also be identified in *CDKN2A* mutation carriers which influence pancreatic cancer risk. It is however unlikely that these variants will be the same as those which influence melanoma risk, as Wu and colleagues demonstrated [13]. Other genetic variants should thus be considered when studying the risk of pancreatic cancer in *CDKN2A* mutation carriers. Yang et al. [14] made a first attempt at this by sequencing the *PALB2* gene in a small cohort of *CDKN2A* mutation carrying families with pancreatic cancer. The *PALB2* gene (OMIM #610355) is one of the relatively few known high risk pancreatic cancer susceptibility genes associated with familial pancreatic cancer to date [15]. However, no pathogenic mutations were identified in their study and also no association was found between SNPs in the *PALB2* gene and the occurrence of pancreatic cancer.

The strength of this study is that our cohort of p16-*Leiden* mutation carriers is homogeneous regarding the type of mutation, and therefore relatively large in its kind. Yet, in order to study modifier effects in a cohort of individuals with a genetically inherited predisposition to cancer, a large sample size is important [16]. Because the p16-*Leiden* mutation is a rare mutation, an important limitation of this study is that sample size cannot easily be increased. Therefore, the lower age limit of the control group was set at 55 years (the mean age of pancreatic cancer in the case group). It is possible that individuals in the control group will develop pancreatic cancer in the future and this could have influenced the results. Possibly, recalculations in the future, taking into account new clinical follow-up data of these individuals, could change the results significantly.

For this study, we selected seven promising pancreatic cancer-associated SNPs from the literature, based on reported odds ratios, p-values and reproducibility. The sample size limitation allowed us to test only a restricted number of SNPs and because of the limited sample size we chose for those with a relatively high minor allele frequency. Our selection of seven SNPs is however only a subset of a much larger set of SNPs associated with pancreatic cancer and it is therefore still possible that these other SNPs do play a role in the pancreatic cancer risk in p16-*Leiden* germline mutation carriers (see [17] for an overview of associated SNPs). It is also possible that other, non-genetic, risk factors play a role in the variable pancreatic cancer phenotype. However, we do not have sufficient data available of these external risk factors in our studied mutation carriers. One of the most important non-genetic risk factors for developing pancreatic cancer is tobacco use, and even in *CDKN2A* germline mutation carriers it increases the risk for pancreatic cancer significantly [18, 19].

## Conclusions

This study aimed at identifying genetic modifiers that influence pancreatic cancer risk in a homogeneous cohort of Dutch p16-*Leiden* germline mutation carriers. Despite the fact that no significant association could be found for the seven tested pancreatic cancer-associated SNPs, it is still possible that these or other genetic modifiers play a significant role in the individual pancreatic cancer risk in these individuals. The search for genetic modifiers for pancreatic cancer in p16-*Leiden* germline mutation carriers should therefore continue.

## Abbreviations

CDKN2A: Cyclin-dependent kinase inhibitor 2A
FAMMM: familial atypical multiple mole melanoma
GWAS: Genome-Wide Association Study
MAF: minor allele frequency
SNP: single nucleotide polymorphism
